# (De)constructing ‘therapeutic itineraries’ of hypertension care: A qualitative study in the Philippines

**DOI:** 10.1016/j.socscimed.2021.114570

**Published:** 2021-11-17

**Authors:** Jhaki A. Mendoza, Gideon Lasco, Alicia Renedo, Lia Palileo-Villanueva, Maureen Seguin, Benjamin Palafox, Arianna Maever L. Amit, Veincent Pepito, Martin McKee, Dina Balabanova

**Affiliations:** a College of Medicine, University of the Philippines Manila, Manila, 1000, Philippines; b Department of Anthropology, University of the Philippines Diliman, Quezon City, 1111, Philippines; c Centre for Global Chronic Conditions, London School of Hygiene and Tropical Medicine, London, WC1E 7HT, UK; d College of Medicine, University of the Philippines Manila, Manila, Philippines; e School of Medicine and Public Health, Ateneo de Manila University, Pasig City, Philippines

**Keywords:** Hypertension, Chronic disease, Patient pathways, Therapeutic itineraries, People-centered care, People centered health systems, Philippines

## Abstract

Hypertension, a major risk factor for non-communicable diseases, remains poorly controlled in many countries. In the Philippines, it is still one of the leading causes of preventable deaths despite the accessibility and availability of essential technologies and medicine to detect and treat hypertension. This paper characterizes the ‘therapeutic itineraries’ of people with hypertension from poor communities in rural and urban settings in the Philippines. We employ longitudinal qualitative methodology comprised of repeat interviews and digital diaries using mobile phones from 40 recruited participants in 12 months. Our findings demonstrate that therapeutic itineraries, rather than being organized according to categories that stem from the structure of the health system (i.e., diagnosis, treatment, follow-up, adherence), diverge from clinical pathways. Therapeutic itineraries begin at a stage we label as ‘pre-diagnosis’ (PD). Following this, itineraries diverge according to two possible entry points into the healthcare system: via incidental diagnosis (ID) whereby participants were diagnosed with hypertension without deliberately seeking care for hypertension-related symptoms and symptom-driven diagnosis (SD) whereby their diagnosis was obtained during a clinical encounter specifically prompted by hypertension-related symptoms. Participants whose itineraries follow the SD route typically oscillated between periods of regular and intermittent medical treatment, while participants who were diagnosed incidentally (ID) typically opted for self-care As we follow our participants’ therapeutic itineraries, we explore the confluence of factors informing their care journey, namely, their conceptions of hypertension, their social relationships, as well the choices and trade-offs they make. We conclude with policy implications from our findings, chief of which is our proposition that models of care based on mere access and availability of clinical interventions fail to reflect the complexity of people’s lay understanding and their lived experiences of hypertension and are thus ultimately unhelpful in improving its control.

## 1 Introduction

Non-communicable diseases (NCDs) are a growing global health issue, particularly in low- and middle-income countries (LMICs) (WHO, 2014). In this paper, we turn our attention to hypertension, a major risk factor for NCDs and leading preventable cause of illness and premature death worldwide. Although hypertension is easily detected and treated with widely available technologies and medicines, it remains poorly controlled everywhere, especially in low-income countries (Chow et al., 2013; Geldsetzer et al., 2019; Irazola et al., 2016; Murray et al., 2020; WHO, 2021).

Concerns about the poor control of hypertension have been supported by a growing number of qualitative studies on the knowledge and attitudes of people with hypertension about their condition. These studies have shown how people’s perception of the disease as an asymptomatic condition and their concerns about the side-effects of long-term medication use often contributed to poor hypertension control (Marshall et al., 2012; Najimi et al., 2018; Najjuma et al., 2020; Shamsi et al., 2017; Tan et al., 2017). Taken together, these studies problematize lay understandings of hypertension as cultural barriers that, alongside health care system barriers, have led to poor health outcomes (Najimi et al., 2018; Risso-Gill et al., 2015; Sison et al., 2020).

In recent years, there has been increasing recognition that understanding ‘patient pathways’ by privileging the factors affecting people’s decision-making process throughout the continuum of care, from initial diagnosis and initial treatment throughout follow-up and adherence, is key to improving effective response to the poor control of hypertension (Brathwaite et al., 2020). One of these studies of patient pathways conducted in Bangladesh showcased those barriers to diagnosis and continuity of care that contributed to poor hypertension control, in particular, are long waiting lines in clinics and inadequate supply of medicines in government health facilities (Naheed et al., 2018).

The anthropological and sociological literature has long been intrigued by people’s management of chronic diseases that require lifelong treatment, providing deeper theoretical and methodological constructs to investigate the complexities of the chronic illness experience. For instance, several of these studies have interrogated the authoritative nature of biomedicine in establishing metrics of ‘good control’ and ‘adherent’ or ‘compliant ‘patients, often overlooking people’s subjectivities, agency, and nuances of their lived experiences in managing a chronic condition (Ferzacca, 2000; Kaljee and Beardsley, 1992; Maskovsky, 2005; Sarradon-Eck et al., 2010; Trnka, 2014; Whitmarsh, 2009). The act of diagnosis as a hegemonic tool of biomedicine to assert the presumed responsibilities of *patients* has also been examined in the ethnographic literature (Manderson, 2020; Rubinstein and Sakaki- bara, 2020; Smith-Morris, 2015, 2020). For instance, Smith-Morris (2020) illustrated how there can be varying degrees of acceptance of a clinical diagnosis: people can partially embrace a diagnosis or choose not to accept it, which can affect their adherence or ways of conforming to the biomedical treatment prescribed (Smith-Morris, 2020). Fortin et al. (2013) showed how various care pathways, each with their own set of actions, emerge to deal with the ongoing medical uncertainties of living with a symptomatic chronic condition amidst a clinical diagnosis with a prescribed treatment regimen (Fortin et al., 2013). Still, structural and political forces are at play, with those hindering access to care being detrimental, especially for marginalized populations experiencing NCDs, trapping them inside an unending loop linking chronic illness and impoverishment, in the process furthering the chronicity of illness and suffering (Manderson and Warren, 2016; Whyte, 2012, 2016).

In this study, we hope to contribute to our understanding of chronic illnesses by contextualizing hypertension care in relation to the temporality of living with hypertension. We draw inspiration from Gerhardt (2006) concept of *itineráries terapêuticos* (therapeutic itineraries) (Gerhardt, 2006) to examine the care itinerary of selected hypertensive individuals in the Philippines. Given the asymptomatic nature of hypertension, imbued with its own diagnostic uncertainties, the language of ‘itinerary’ provides us a framework to better understand people’s decision whether to engage or disengage with several care practices including biomedical and clinical care, self-care, and alternative forms of treatment.

### 1.1 NCD control framework in the Philippines

The Philippine Department of Health’s NCD control program, known as PhilPEN (the Philippine Package of Essential NCD interventions) (DOH, 2012), largely follows WHO’s prescriptions for NCDs, which emphasize primary health care and integrated approaches to affect behavioral changes intended to mitigate hospital-centered acute care where people only seek help when they have experienced complications. Under PhilPEN, standardized primary health care management, particularly for diabetes and hypertension, ensures early detection and use of cost-effective procedures for diagnosis and treatment are implemented at the lowest levels of the health system. Once diagnosed, individuals are invited to participate in local ‘hypertension and diabetes clubs’, which provide free medicines, healthy lifestyle counseling, as well as outpatient services (e.g., blood pressure monitoring) in government-run health clinics. However, these government-mandated clinical guidelines may not reflect the processes operating in the private health sector, which is predominantly market-oriented and where healthcare services medications, and other interventions at all levels come at a cost (Dayrit et al., 2018; DOH, 2005). Efforts to manage NCDs are complicated by the decentralized public healthcare system that has resulted in fragmented healthcare delivery (Dayrit et al., 2018; Pinlac et al., 2015; Ulep et al., 2020).

Recent legislative measures in support of lifestyle and behavioral changes have been accompanied by declining trends in smoking and alcohol consumption (Dayrit et al., 2018). In addition, the government has recently passed a Universal Health Care Act, with the stated aim of ensuring and improving access to health care for all Filipinos, where health needs are met at the appropriate level of care. Despite these measures, the prevalence of hypertension continues to increase and NCDs are forecasted to pose an increasing burden in the coming years (Ulep et al., 2020).

Further undermining NCD management in the country has been the lack of a robust and clear referral system to higher-level health facilities and back to primary care (Dayrit et al., 2018; Ulep et al., 2020) as well as irregular monitoring and inadequately sustained implementation of efforts on the ground (Pinlac et al., 2015). Such deficiencies are particularly catastrophic for low-income households suffering from NCDs as half of the Philippine population – many from poor communities–bypass primary care and directly engage with tertiary hospitals for acute care, rendering them vulnerable to financial costs (Ulep et al., 2012, 2020). Simultaneously, the Philippine health system faces other systemic issues, including geographic maldistribution of health facilities and human resources with heavy concentrations in urban localities, increasing out-of-pocket health expenditures, and an expanding, well-resourced, and high-quality private health sector (Dayrit et al., 2018). It is projected that the number of Filipinos with hypertension will continue to rise from 14 million in 2020 to 30 million by 2040. Hence, local public health experts have called for the strengthening health system responses, developing multisectoral plans for NCD prevention and control, and employing integrated “whole-of-society” and “whole-of-government” approaches (Castillo-Carandang et al., 2020; Pinlac et al., 2015).

Most hypertension management studies in the Philippines have focused on clinical outcomes and the biomedical aspects of treatment and adherence, with only a few examining the social and cultural dimensions of hypertension care (Lasco et al., 2020; Rueda-Baclig and Florencio, 2003). We argue that poor blood pressure control could be better understood if the analytical tools and concepts used to map and control a disease are grounded more firmly in people’s life circumstances and belief systems. We move away from pre-determined clinical stages (e.g., diagnosis, treatment, follow-up, and adherence), and instead, we privilege the emic perspective of the individuals. We do this by characterizing the therapeutic itineraries of people with hypertension in rural and urban settings in the Philippines by drawing on the analysis of data from repeated qualitative interviews and ‘digital diaries’ which allowed our hypertensive participants to share their daily experiences of managing their condition. In the process, we focus our attention on the confluence of factors affecting people’s care journey, namely the temporality of hypertension, its sociality, and the trade-offs that people make.

## 2 Methods

### 2.1 Data collection

This study was conducted as part of the (details omitted for blind reviewing) project, which examines ‘patient pathways’, and barriers to hypertension care in the Philippines and Malaysia using mixed-methods (Palafox et al., 2018). In this paper, we draw on the data generated from the qualitative component. The study was conducted on the island of Luzon, Philippines, with Quezon Province as the rural site and Valenzuela City in Metro Manila as the urban site. 620 participants were randomly selected to participate in a quantitative household survey, who were either ‘aware’ hypertensives that had received a clinical diagnosis previously, or ‘unaware’ hypertensives who were screened and found to have high blood pressure during the household survey. The qualitative component purposively selected from the quantitative cohort of participants 40 individuals according to age, sex, type of residence (urban/rural), and awareness of their hypertension (See Table 1). The initial plan was to recruit a sample of participants balanced by sex and age. However, most of the participants who agreed to participate when contacted by the researchers were female and of the older age group, who were primarily homemakers as they were the ones usually at home. Those from the urban areas were mostly informal and contractual workers who were daily wage earners (e.g., drivers, vendors, house help and factory workers). Four of our respondents were working as Barangay (Village) Health Workers during the data collection phase. Those in the rural areas were mainly farmers and fisherfolk who also worked on a daily basis. Following the quantitative survey, local researchers contacted the selected participants by phone to confirm their interest to participate in the qualitative component of the project and to schedule a visit. Interviews were mostly conducted in participants’ homes or in neighbourhood spaces, such as community gymnasiums and nearby health centers. We scheduled interviews according to the dates that our participants identified as most convenient for them. Some interviews were conducted on weekends or on a weekday afternoon to accommodate workers. Data were collected between October 2018 and November 2019.

### 2.2 Data analysis

We draw on 71 semi-structured interviews with hypertensive individuals (40 initial, 31 after 12 months follow-up). The first and second authors conducted the interviews, which lasted from 45 to 60 min, in Filipino (Tagalog) and focused on people’s illness narratives, perceptions of hypertension, and experiences with the health care system. In the period between the initial and follow-up interviews, respondents were asked to complete digital diaries using mobile phones to track their lived experiences of hypertension for a year. In doing so, we hoped to capture information on their everyday lives and on the social, economic, and cultural factors that could affect their care journeys. Digital diary entries were stored, accessed, and managed by the researchers using a secure web-based platform that allowed concurrent analysis. It is important to note that participants engaged with the digital diaries at low levels and did not provide the in-depth narratives expected by the researchers. The majority of the digital diaries contain only nominal responses about hypertension symptoms and treatment. This is likely related to the largely asymptomatic nature of hypertension, which limited the participants’ physical experience of the illness. Also, many did not perceive hypertension to be a serious or chronic condition requiring constant attention, partly reflecting local conceptions of the condition (Lasco et al., 2020). Finally, technical limitations of the platform only allowed participants to submit short message services (SMS) text message diary entries. Although the digital diary platform was developed to capture entries in multiple formats submitted via mobile networks, including voice and text messages, photographs, and videos, the network servicing the platform in the Philippines only permitted SMS text messages. Some respondents overcame this limitation by submitted entries in other formats, such as audio messages via Facebook messenger; some older participants were also less comfortable using mobile phones. More in-depth analysis of our experiences using the digital diary platform, and its weaknesses and strengths as a method for data collection and aggregation is published elsewhere. We recommend that digital diaries can complement other qualitative methods, such as in-depth interviews, which should allow a more comprehensive tracing of participants’ itineraries (Mendoza et al., 2021).

All interviews and digital diary entries were transcribed in Filipino and translated to English, given the international composition of the research team. The first author led this process with the help of other local research team members and worked with the other co-authors on conceptual framing and coding. For the first analytic step, the first and second authors used the original Filipino transcripts to characterize the therapeutic itineraries of participants using the emic (i.e., participants’ own) perspective. Guided by principles of grounded theory (Charmaz, 2006; Strauss and Corbin, 1997), the interviews (as the primary source of data) and digital diary entries (as supporting data) were inductively coded and thematically analyzed (Thomas, 2006) using NVivo software (Jackson and Bazeley, 2019). Our use of grounded theory at this step sought to avoid the imposition of a clinically-driven framework on the characteristics of the participants’ therapeutic itineraries, while also ensuring reflexivity in the analytical process (Finlay and Gough, 2003; Hennink et al., 2011). The second analytic step sought to characterize and refine each of the stages identified in the pathways by plotting the therapeutic itinerary of each of the forty participants on a spreadsheet. During this process, we paid attention to participants’ movements along the pathway from diagnosis to care, and how movements or points of change (i.e., in health-related practices or care accessed) were related to other socio-cultural, personal, or structural factors. Based on individualized pathways, a coding framework was developed collaboratively by the broader research team, comprised of local and foreign researchers, all of whom participated in the plotting, coding, and ultimately, identification of the themes through consensus.

Ethical approval for the study was granted by the London School of Hygiene and Tropical Medicine Observational Research Ethics Committee and the University of the Philippines Manila Research Ethics Board Panel 1 (Ref: 2017-481-01).

Written informed consent was provided by all participants before our data collection.

## 3 Findings

### 3.1 Entry points

**Fig. 1:**
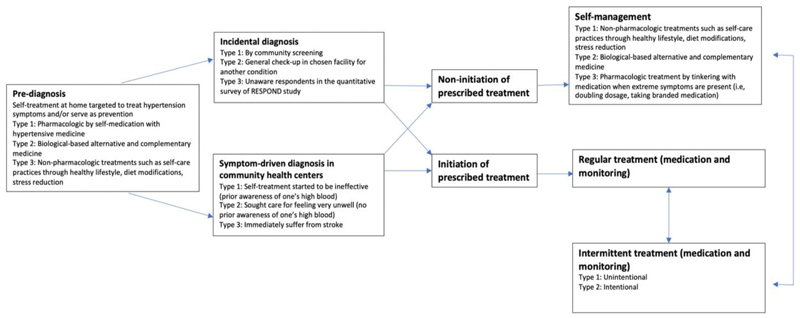
Movement of people in their therapeutic itineraries.

‘Therapeutic itineraries’ (see Fig. 1) begin at a stage we label as ‘pre-diagnosis’ (PD). Following this, itineraries diverge according to two possible entry points into the healthcare system: via incidental diagnosis (ID) whereby participants were diagnosed with hypertension without deliberately seeking care for hypertension-related symptoms and symptom-driven diagnosis (SD) whereby their diagnosis was obtained during a clinical encounter specifically prompted by hypertension-related symptoms. Participants whose itineraries follow the SD route typically oscillated between periods of regular and intermittent medical treatment, while participants who were diagnosed incidentally (ID) typically opted for self-care.

Participants constructed their self-care regimes based on their bodily symptoms. Even before any engagement with the healthcare system, the ‘pre-diagnosis’ phase featured several self-care actions intended to prevent, treat and manage their high blood pressure, commonly referred to in the Philippines as ‘high blood’/*‘mataas ang presyon’/‘presyon’* (Lasco et al., 2020) and typically self-diagnosed based on certain symptoms, such as pain in the nape of the neck, headache, and fatigue, which was validated or confirmed via informal blood pressure measurements. Some study participants had access to a blood pressure monitoring device in their homes (these are usually manual or digital rubber cuff), either purchased or gifted to them; others borrowed them from their neighbors or obtained measurements from Barangay Health Workers (BHWs).

Initial forms of self-care treatments included both pharmacological and non-pharmacological modalities, such as self-medication with conventional antihypertensive medicines, herbal remedies, taking periods of rest and relaxation, and introducing lifestyle changes, especially in their diet. Social circles, both virtual and physical, were a primary source of knowledge on self-care practices for participants.

This cycle of home-based self-care is the typical first-line therapeutic strategy used to relieve what are believed to be hypertension-related symptoms. The decision to finally consult a conventional doctor typically arises when such first-line strategies cease to relieve the symptoms of ‘high blood’, leading to the receipt of a ‘symptom-driven diagnosis’ of hypertension.

Incidental diagnoses of hypertension, on the other hand, were obtained in several ways, including routine company check-ups, casual consultations at health centers, and instances where a family member measured the participant’s blood pressure or brought them along to a clinic. Some respondents were screened for hypertension because of membership in the *Pantawid Pamilyang Pilipino Program* (4 P), a national conditional cash transfer program of the Department of Social Welfare and Development for indigent families that includes monthly health check-ups of recipients. We also identified several participants who were ‘unaware’ of their condition (i.e., had not previously been diagnosed with hypertension by a health professional), but who still engaged in preventative self-care as they were not yet experiencing any symptoms.

Narding (60-year-old retired factory worker from Valenzuela, hypertensive for 10 years) who was initially diagnosed with hypertension during a company check-up, was not convinced of his diagnosis mainly because he had not experienced bodily symptoms: *“There were no signs. I* was *provided with medicine, but I didn’t take them…. I sold it to others who like to take medicine instead”.* After several years, he developed bodily symptoms associated with hypertension, sought medical attention, and by the time of the interview had accepted his clinical diagnosis of hypertension. He reported taking antihypertensive medication at the time of the interview but also confided that he intentionally stopped treatment when symptoms subsided due to concerns about side-effects which he had heard about from others.

Most participants who were ‘unaware’ of their hypertension at the initial interview did not report initiating any types of formal care during their follow-up interviews or digital diaries and only practiced self-care. In addition to the absence of symptoms, these participants mentioned that they were too busy with work to seek consultation. Fred, a 61-year-old farmer from Quezon Province, shared during his initial interview that the lack of symptoms was the reason for deciding not to seek care: *“Nothing. I just brushed it off. Sometimes you get ‘high blood’, you get ‘low blood’. But that day during the survey, I didn’t think I had [high blood] pressure. There was nothing. I wasn’t feeling anything.”* After a year, during his follow-up interview, he shared: *“I haven’t had any check-ups yet, even now because I don’t feel anything. And, I don’t have the time yet because I need to work. It will just be an interruption. Besides, my only concern is body pains, and I take mefenamic [acid, an analgesic] for that.”*

In the following sections, we discuss the factors underpinning people’s therapeutic itineraries for hypertension, namely the temporal dimension of hypertension, people’s social relationships and their socioeconomic realities, and how these influence provider choice.

### 3.2 Temporality of hypertension

Once individuals were clinically diagnosed, many initiated regular treatment through frequent blood pressure monitoring and by taking the medication regularly as prescribed. Eventually, they managed their condition intermittently contrary to medical advice. In their explanations for this intermittent approach, they alluded to the asymptomatic nature of hypertension and to their non-chronic and time-bound view of the condition, an illness that *“just comes and goes”,* which led them to either unintentionally or intentionally discontinue their prescribed medication because they felt better – even despite having been given medical advice on the importance of regular and continuous adherence. The sporadic booking of follow-up appointments and the resumption of regular medication and blood pressure monitoring were usually prompted by the recurrence of persistent and/or extreme bodily symptoms.

As illustrated in Fig. 1, we encountered several instances among those who received a symptom-driven diagnosis who opted not to initiate treatment. Again, a belief in the non-chonicity of hypertension appears to determine this choice, as does the view of the condition as ‘natural’. Aida (a 60-year-old homemaker from Quezon, hypertensive for 3 years), explained, *“I was experiencing headache and nape pain. I went to the health center and my blood pressure* was *checked by the BHW. It was 140 [mmHg]. The BHW told me to get maintenance [medication]. But I didn ’t take them … because it’s natural. High blood pressure happens … and ‘maintenance’ means I have to maintain taking it, right? I don’t want that. I don’t want to take medicine because eventually, I will forget about it. Besides, sometimes it’s there, but eventually, it will disappear.”* Nancy (49-year-old homemaker from Valenzuela City, hypertensive for 5 years) echoed the same belief about the inevitability of hypertension and its curable and non-chronic nature: *“That’s just normal in life. It can happen as you grow older. If you can fight it off with a diet that’s better because then it will not come back.”*

A few participants reported how their condition was rapidly followed by a stroke, despite having regularly adhered to their antihypertensive medication regime; while two participants on the symptom-driven diagnosis pathway experienced a stroke after adhering only intermittently to their prescribed treatment. Julian (50-year-old retired school bus driver from Valenzuela City, hypertensive for 5 years) shared, *“That’s why I am like this now [laughs]. I am paralyzed because I was not able to take the medicine regularly, sometimes I stopped. I used to forget and sometimes when I feel ‘high blood’ back then my body was able to manage. So now, I really take my medicine now every day. It’s important. Both in the morning and afternoon.”*

### 3.3 Relationality and sociality of hypertension

We found that participants’ social relationships were entangled within their therapeutic itineraries, which participate in the co-construction of care trajectories, reflecting the relational and social aspects of the disease experienced by those with chronic conditions (Gerhardt, 2006; Nichter, 2002; Rubinstein and Sakakibara, 2020; Trad et al., 2010; Whyte, 2012). Participants’ social circles were primary sources of information for various forms of treatment, both pharmacological and non-pharmacological, such as the types of food, herbal supplements, and medications that are good for high blood pressure. Some participants ‘became’ chronically ill by extension just from being aware of a family history of hypertension and out of fear from the risk of future serious complications – particularly if family members had suffered from comorbid events, such as stroke. Shared chronic management experiences within the family led some participants to purchase a blood pressure monitoring device and learn how to incorporate self-monitoring into their lives, even before receiving a clinical diagnosis or without experiencing bodily symptoms for hypertension.

Dennis a 64-year-old farmer in Quezon Province shared, *“I have a history. My father had a stroke back then, and you know, most of us in the family were involved in taking care of him, especially us, his children. He also has ‘high blood’. So, my wife usually checks my blood pressure, then all of sudden I was getting high blood pressure readings – 140, 150 [mmHg]. So, I thought to myself, ‘this is strange’ and ‘this might be something serious’. But I wasn’t feeling anything. My wife also urged me to get a check-up with a doctor.”*

Daisy, a 42-year-old homemaker in Quezon Province with hypertension for 5 years, shared a similar experience of how she discovered her high blood pressure in the absence of bodily symptoms, *“I am also afraid, you know, of the possible complications from ‘high blood’. I know it can be difficult. My father, mother, and siblings had strokes. So I learned how to occasionally check and monitor my blood pressure.”* This prompted her to try a medication suggested by one of her peers that cause undesirable side-effects, which contributed to her decision to finally see a doctor.

Indeed, family members (particularly spouses and children) and social networks play a prominent role throughout the therapeutic itinerary. For instance, they reminded participants to take their medication, collected free medicine from health centers on their behalf, and encouraged them to seek clinical care or pursue self-care. Also evident is how participants’ children can take charge of their parents’ health expenses and decision-making, becoming key actors in their ‘therapy managing group’ (Janzen, 1987). When children had control over their parents’ disease management, participants were more prone to following regular treatment. Moreover, participants’ motivations to start adhering to prescribed treatment regimen are driven by familial affection and what Varul calls “the guilt anxiety of letting down dependent family members” (Varul, 2010, p.89). This highlights how the care process is not only produced by individuals for their own sake but rather how it is relational and co-constructed with and against significant others. Narciso (53-year-old factory worker from Valenzuela City, hypertensive for 14 years) shared: *“I really monitor myself for my children. I don’t want them to experience what I went through when I was taking care of my parents who were also ‘high blood’. And now I am taking care of my brother who has it too and had a stroke.”*

Daisy (44-year-old homemaker from Quezon Province, with hypertension for 5 years) shared similar insights: *“I really think regular medication is important. It’s not just for me, but also for my kids. What will happen to them in case, you know? So, I try to take the medicine regularly. But honestly, I keep on forgetting.”*

### 3.4 Precipitating trade-offs

After diagnosis and treatment initiation, the inconvenience of traveling to seek care and long waiting times at facilities led participants to make trade-offs, posing a threat to achieving optimal hypertension management and control. For instance, these trade-offs included prioritizing work over going for check-ups or laboratory examinations. For those with day jobs, clinic hours and doctors’ availability did not typically align with their periods of free time. Participants also reported that free medication for hypertension, collected monthly, was not always available at health centers. This led some either to a pattern of intermittent adherence or to incur out-of-pocket expenses to purchase their medication from private outlets. When asked about the financial burden posed by such medicine stockouts, many shared that the impact was small as they only purchased a few tablets at a time to last for 1–2 weeks, but that doing so could also result in intermittent adherence.

Moreover, many of our participants alluded to the role of convenience in continuing care. For example, Marie shared in her digital diary, *“I stopped with my medicine for a month now. I now grow ‘serpentina’ leaves at home and make tea out of them. I feel better with herbal.”* In subsequent exchanges with her by phone, she added, *“The herbal is readily available at home, which is more convenient for me because I can’t find the time to go to the health center for the free medication and they are only open on weekdays. My free time is on weekends.”* Other participants had similar reasons for shifting to a different therapeutic strategy, alluding to convenience factors as reported in their digital diary entries and follow-up interviews.

Many respondents also recognized the importance of regular medication but were ambivalent about its ongoing and long-term use. They shared concerns about the possible side effects and the possibility that their bodies could get “used to” medicines. As a compromise, some of them deliberately took medication breaks and/or shifted to herbal remedies periodically. Others used herbal remedies concurrently alongside conventional medication to counter possible side-effects from the latter. Participants viewed their conventional medication as a commitment that can be renegotiated when any hypertension-related symptoms subside, which itself is based on the belief that they are able to assess the needs of their own bodies. In addition, younger participants felt that regular medication may not be necessary, saying that their bodies were capable of ‘withstanding’ hypertension symptoms. Meanwhile, others have shared the belief that regular hypertensive medication can be excessive at times and can lead to ‘low blood pressure’, which was yet another reason to suspend pharmacotherapy. Clearly, people’s concerns about medication and their sense of agency in deciding what they perceived as best for their bodies influence their decisions to either engage or disengage from the formal care process.

### 3.5 Choice of healthcare provider

In both urban and rural settings, participants’ choice of health facility when deciding to seek care was primarily influenced by convenience, in terms of their time, budget and distance, given that most work daily. Most used local health centers because they offer free primary care services and are present in every barangay (village, the smallest administrative division in the Philippines), even though they frequently experience long waiting times. For those with sufficient financial resources to pay for and/or social capital to readily access private sector care, only distance was the primary factor when choosing their preferred provider.

Valenzuela City, our urban study site, had more and a wider variety of health facilities available to choose from. Aside from the barangay health centers and public sector hospitals, private clinics, hospitals, diagnostic centers, and pharmacies also offer primary care services. Facilities in neighboring areas within the Metro Manila region were also accessible. In contrast, fewer and only public facilities were available in our rural sites across Quezon Province, where participants who were able to access private care must travel one to 2 h to reach their preferred provider. In Quezon, municipalities typically operate a single tertiary hospital, apart from one island municipality included in our study where our participants must travel by boat to the mainland for specialist and emergency care.

As for the use of traditional, complementary, and alternative medicine options for hypertension, there were no clear differences between the rural and urban sites. Many participants occasionally relied on herbal remedies to manage their hypertension, and the choice to do so tended to be influenced more by popular culture and knowledge, rather than through consultation with folk healers. Also, hypertension was perceived by most as a condition best treated by practitioners of biomedical, rather than traditional, medicine, even among those who used both modalities.

## 4 Discussion

Our findings demonstrate that therapeutic itineraries — shaped by people’s own lived experiences, understanding of care, and beliefs about what constitutes hypertension, rather than being organized according to categories that stem from the structure of the health system — diverge from clinical pathways. People construct their own systems of care in ‘cooperation with’ bodily symptoms and significant others, both playing an important role in movements along their illness trajectories. ‘Diagnosis’, for instance, can be seen as a natural first step in a clinical pathway and a beginning of a journey; but even prior to any engagement with the health care system, the ‘pre-diagnosis’ phase features a number of health-related decisions and actions targeted to prevent, treat and manage their high blood pressure, characterized by experiencing bodily symptoms. This is consistent with earlier studies on care pathways and therapeutic itineraries (Fortin et al., 2013; Gerhardt, 2006; Trad et al., 2010), wherein self-care with conventional and traditional forms of medicine emerged as the first-line of therapeutic care to solve a ‘mild’ health problem at hand. Seeking care from a different source, for instance, the biomedical health system, comes as a second option when self-care starts to be ineffective, as seen with a few of our participants who sought care for the unbearable symptoms they felt. For those who were incidentally diagnosed, not only the lack of symptoms but also their need to prioritize work over seeking care provides context for their ‘inaction’. This intimate relationship between how a person self-assesses being healthy or not in relation to one’s capability to work was also observed in another study of therapeutic itineraries on chronic conditions among poor households (Burille and Gerhardt, 2014).

Clearly, one of the challenges in addressing chronic NCDs is their relative ‘invisibility’: that is, they are oftentimes barely perceived by people because the onset and magnitude of their symptoms are not dramatic compared to acute and infectious diseases (Manderson and Smith-Morris, 2010). Consequently, people described a lack of seriousness, concern, and urgency with which hypertension is viewed in our study and corroborated in others (Chow et al., 2013; Marshall et al., 2012; Risso-Gill et al., 2015), especially in relation to other conditions like cancer, an observation also documented in other settings (Drew and Schoenberg, 2011; Livingston, 2012; Lora-Wainwright, 2013). Dominant public health interventions for NCDs, largely drawn from global health models, favor access and availability through primary health care interventions, risking the quality of care because they are often configured in a standardized manner (Kleinman and Hall-Clifford, 2010). In this biomedical framing, people are expected to be responsible and disciplined agents creating only adherent or non-adherent patients or compliant or non-compliant patients. But this “problem of compliance” (Ferzacca, 2000) to biomedical and clinical pathways is ever-present for NCDs as people generally privilege their subjectivities and embodied realities of these conditions affecting how they engage or disengage from the treatment process (Sarradon-Eck et al., 2010; Trnka, 2014; Whitmarsh, 2009).

Our findings point to a confluence of factors that influence people’s therapeutic itineraries for hypertension resulting in different ways of obtaining and maintaining control in hypertension. People’s ‘lay physiologies’ are among the primary drivers of their ways of seeking control. In our previous study, we identified how the folk physiology of hypertension that rests on the local understanding of changes in blood and blood flow attributed to genetics, stress, heat, and diet, contributed to the fluctuations of blood pressure (Lasco et al., 2020). This provides the rationale for their non-chronic or temporal view of hypertension, an illness that comes and goes, resulting in their sporadic use of prescribed treatment, which may be intentional or unintentional. Unless a more serious condition develops, for instance, a stroke, only a dramatic change in their bodies may prompt them to fully commit to the biomedical treatment, as also supported by a systematic review on hypertension pathways (Brathwaite et al., 2020). Once they are diagnosed, people show remarkable agency in their self-management as they bear the final responsibility and consequences in deciding what is best for their bodies. As with asthma management (Trnka, 2014), we have seen how people ‘move in and out’ of hypertension, which could also be understood in the context of partially embracing a diagnosis (Smith--Morris, 2020). This may also help to explain described instances of intermittent adherence to biomedical treatment or the preference for self-care as a rejection of a clinical diagnosis. In addition, our findings also revealed that there are concerns about the side-effects from long-term use of medication, either subjectively felt or heard from others, resulting in intentional medication breaks for some and shifts to alternative and complementary medicine for others. Symptoms serve as people’s individualized warning signs (Trnka, 2014) of how they choose to self-manage their condition. And in the absence of symptoms, disengaging from their medication and the preference for self-care is seen as a rational step in the care process. But despite some people’s belief in their perceived ability to monitor and predict high blood pressure through their bodies, many still perform routine blood pressure checking using monitoring devices, which also reflects the importance of biomedical technology in confirming their felt realities. Knowing about their family history of hypertension and having an actual experience of taking care of a family member who suffered from stroke linked to hypertension created a vicarious chronic experience for some of our participants, which prompted them to monitor their blood pressure despite the lack of symptoms. Clearly, care is a matter of “tinkering” and “attentive experimentation” for people with chronic conditions (Mol et al., 2015), in the process, resulting in hybrid forms of enacting and resisting biomedical forms of care and active blending of other forms of treatment, such as self-care and alternative medicine (Ferzacca, 2000; Smith-Morris, 2020).

Nonetheless, despite the perceived active role of our participants in deciding the best course of treatment for their bodies, systemic challenges also affect their ways of obtaining and maintaining control that oftentimes discourage continuity of the prescribed treatment and monitoring for hypertension, as also observed in other settings (Nyaaba et al., 2018; Whyte, 2016). Our study participants in both sites still encounter structural barriers, such as lack of facilities necessitating long-distance travel to access care, long waiting lines in clinics which can discourage them to engage with the health system and insufficient supply of medication in health centers which can lead to out-of-pocket expenses, reflecting the deficiencies and health disparities in the Philippine health system (Dayrit et al., 2018; Ulep et al., 2012, 2020). Such challenges have also made continuity of care for NCDs more challenging for our poor urban and rural participants alike, perpetuating the phenomenon of “recursive cascades” between chronic ill health and poverty (Manderson and Warren, 2016). And despite the encouragement of preventative care and self-management among health providers, access to essential medical technology, such as blood pressure monitoring device, is still not widespread. As seen in our findings, some of our participants rely on their neighbors and BHWs to check their blood pressure, and their social circles are primary loci of knowledge circulation on self-care practices. Indeed, social dynamics permeate and contribute to people’s construction of their journeys to care. Similar to other studies on hypertension management in LMICs (Abbas et al., 2020; Chacko and Jeemon, 2020; Costa and Nogueira, 2008; Hu et al., 2015; Marshall et al., 2012; Trad et al., 2010) we see a positive association between family social support and hypertension self-care.

Primary care in the Philippines is still largely clinic-based; hence, in order to access hypertensive medication and monitor blood pressure, people must return to clinics, which may not be easy for all due to the trade-offs that people must make (Brathwaite et al., 2020). While the free care and medication offered by public sector health clinics may be readily accessed by some of our participants, such as retirees and homemakers, others, including informal sector daily wage earners who represented a large portion of our sample, tend to prioritize work over attending health centers during their opening hours (Atinga et al., 2018; Nayeri et al., 2015; Risso-Gill et al., 2015). This finding may help to explain why a large share of the Philippine population, particularly the poor, bypass and/or sporadically engage with primary care (Ulep et al., 2020) because they opt for other providers that are more convenient, such as private specialist clinics or because of the need to engage with the health care system only when they experience severe complications. The value attributed to convenience may also be reflected in several other common features of our participants’ itineraries, including the decision to self-monitor one’s blood pressure and self-diagnose when the tools are already in the home during the pre-diagnosis phase, and also the choice to initiate self-care using easily accessible herbal remedies either to complement or as an alternative to their prescribed medication. Interestingly, we also observed how the value attributed to convenience seem to vary depending on the severity of the symptoms or complications being experienced: therapeutic itineraries tended to converge with more clinically defined pathways during periods of greater severity (e.g., better adherence to prescribed medication and more regular attendance to follow up appointments, more engagement with the healthcare system). Moreover, our participants’ considerable agency in the care process could also be understood as a coping strategy as they work to preserve and restore their health in the face of various challenges (Burille and Gerhardt, 2014).

These study findings could help to inform the design of interventions for improving both hypertension and NCD control in the Philippines and other LMICs, and particularly related reforms that seek to make health systems more people-centered. These findings do not seek to make a case for individualized medicine; clinical pathways and guidelines are essential tools for improving the quality of care and optimizing people’s care journeys given the available resources. However, they do suggest that a robust health system capable of addressing the growing burden of chronic NCDs should be adaptive and consider the ever-changing realities of people who require care, and be able to recommend and deliver effective practices that are feasible for them to participate in. Thus, formal pathways must have in-built flexibility to accommodate the specific needs and realities of particular groups, especially those with limited time and resources; for hypertension, this might involve an integrated mix of both community and clinic-based care. Care models should also find ways to better leverage familial and social networks to enhance care quality and outcomes. Such changes likely require changes to the current paradigm that privilege hierarchy and the provider-patient relationship towards one that better accommodates the social realities of patients (WHO, 2015; Sheikh et al., 2014a; Sheikh et al., 2014b). Achieving such a shift will require a collaborative approach that involves not only experts but also grassroots actors, working across the multiple sectors that impact therapeutic itineraries.

## 5 Conclusion

If we are to tackle the persistent problem of poor hypertension control, people’s perspectives and in particular, their logics of care, which are often not incorporated into models of care (Mol, 2008), must be considered. We must rethink how individuals, as people in relation to their felt bodies and significant others, engage with the health system at each step of their journey. For example, ‘incidental diagnosis’ must be recognized both as a challenge and opportunity, mindful that many individuals who enter the system through that route progress no further. Allowing flexibility and space to recognize and build on different trajectories within clinical settings (Fortin et al., 2013; Manderson and Smith-Morris, 2010; Smith-Morris, 2020) can improve outcomes and the ability of the health systems to provide care that is better aligned with the needs of individuals.

Beyond the usual structural barriers, the gap between lay and professional beliefs about the nature of hypertension, one’s ability to assess its effects, and the pathways that are most appropriate to follow should be narrowed instead. This involves, in the first place, understanding local notions of hypertension, chronicity, and risk, and importantly, plotting these notions not only in terms of individuals’ engagement with the healthcare system but in terms of their broader, dynamic practices, which also include self-care and traditional and alternative medicine. Optimal management of hypertension should not be defined as adherence to clinically defined treatment with individuals regarded as mere recipients of care, but rather as involving co-production by working with individuals and communities to create a fruitful therapeutic alliance. Interventions need to span the health system and social spheres, taking full account of people’s therapeutic itineraries as well as the knowledge, experiences, and social realities that shape them.

## Figures and Tables

**Table 1:** Sampling characteristic of our qualitative respondents.

Age group	Urban			Rural			Awareness	
	Female	Male		Female	Male		Aware	Unaware
30–49	7	1		3	2		11	2
50–70	6	7		10	4		22	5
